# Diffuse large B cell lymphoma derived from nodular lymphocyte predominant Hodgkin lymphoma presents with variable histopathology

**DOI:** 10.1186/1471-2407-14-332

**Published:** 2014-05-13

**Authors:** Sylvia Hartmann, Mine Eray, Claudia Döring, Tuula Lehtinen, Uta Brunnberg, Paula Kujala, Martine Vornanen, Martin-Leo Hansmann

**Affiliations:** 1 Dr. Senckenberg Institute of Pathology, Hospital of the Goethe University, Theodor-Stern-Kai 7, Frankfurt am Main D- 60590, Germany; 2 Department of Pathology, Tampere University Hospital and University of Tampere, Tampere 33520, Finland; 3 Department of Oncology, Tampere University Hospital, Tampere 33520, Finland; 4 Department of Internal Medicine 2, Hospital of the J. W. Goethe University, Frankfurt am Main, Germany

**Keywords:** Nodular lymphocyte predominant Hodgkin lymphoma, Diffuse large B cell lymphoma, Transformation

## Abstract

**Background:**

Nodular lymphocyte predominant Hodgkin lymphoma (NLPHL) usually presents in middle aged men and shows an indolent clinical behavior. However, up to 30% of the patients present a secondary transformation into aggressive diffuse large B cell lymphoma (DLBCL). The aim of the present study was to characterize morphology and immunophenotype of this kind of DLBCL in detail and compare it with conventional DLBCL.

**Methods:**

Morphology and immunophenotype of 33 cases of NLPHL with simultaneous or sequential transformation into DLBCL were investigated. These cases were compared with 41 de novo DLBCL in Finnish men.

**Results:**

The majority of cases exhibited different immunophenotypes in the NLPHL and the DLBCL components. The immunophenotype of the DLBCL secondary to NLPHL was heterogeneous. However, BCL6, EMA, CD75 and J-chain were usually expressed in both components (≥73% positive). Overall, the NLPHL component was more frequently positive for EMA, CD75 and J-chain than the DLBCL component. In contrast, B cell markers, CD10 and BCL2, were more frequently expressed and were expressed at higher levels in the DLBCL component than in the NLPHL component. In the independent series of de novo DLBCL 4 cases could be identified with a growth pattern and immunophenotype that suggested that they had arisen secondarily from NLPHL.

**Conclusions:**

The morphology and immunophenotype of DLBCL arisen from NLPHL is heterogeneous. Further characterization of the particular molecular features of this subgroup is warranted to be able to better identify these cases among conventional DLBCL.

## Background

Nodular lymphocyte predominant Hodgkin lymphoma (NLPHL) is a rare subtype of Hodgkin lymphoma (HL), which accounts for approximately 5% of all cases [1] and generally presents with early clinical stages and an indolent clinical behavior [2,3]. Middle aged males account for 75% of cases [3]. Histologically, NLPHL typically consists of large nodules of reactive small B cells with a few intermingled tumor cells, the so called lymphocyte predominant or LP cells. Morphologic variants with a diffuse pattern or an abundant reactive T cell infiltrate mimicking T cell/histiocyte rich B cell lymphoma (THRLBCL) have been described [4,5]. Gene expression profiling studies have identified only marginal differences between LP cells and tumor cells of THRLBCL [6,7]. In contrast to Hodgkin and Reed-Sternberg cells of classical HL, LP cells usually show a preserved or only partially downregulated B cell phenotype [6,8]. They typically exhibit constitutive NF-kappaB activity [6] and active JAK-STAT-signaling [9]. Recently, germline mutations of the *NPAT* gene have been identified as risk factor for NLPHL in the Finnish population [10] and there are reports about a high familial risk in first degree relatives of NLPHL patients [11,12].

In the past 20 years, simultaneous or secondary transformation of NLPHL into diffuse large B cell lymphoma (DLBCL) has been described in up to 30% of cases [13-18]. Several studies have suggested that these patients have a favorable outcome [13,14,16,17,19]. However, one study did not confirm this observation [15]. The male: female ratio for cases of NLPHL transformed into DLBCL was slightly higher than for primary NLPHL (77 - 81%) [14-17]. The aim of the present study was to characterize morphology and immunophenotype of DLBCL simultaneously or sequentially derived from NLPHL in 33 cases. A second aim was the comparison of the immunophenotype observed in these particular cases with conventional DLBCL, in order to identify a marker panel, which allows to detect DLBCL, representing a putative transformation from NLPHL, in an independent series of DLBCL.

## Methods

### Tissue samples

A series of 33 consecutive cases of composite lymphoma of NLPHL and DLBCL were identified from the archives of the Dr. Senckenberg Institute of Pathology, Goethe University, Frankfurt am Main, Germany and the Department of Pathology, Tampere University Hospital, Tampere, Finland. Original diagnoses were made between 1987 and 2013. Original slides were complemented by additional stainings (see below). All diagnoses could be confirmed according to the WHO classification 2008 after joint review (S.H., M.E., M.V. and in part M.L.H.). In 26 cases both the NLPHL and DLBCL were present in the same biopsy, and in 7 cases, the DLBCL occurred sequentially after NLPHL. Two patients with simultaneous presentation of NLPHL and DLBCL had a previous history of NLPHL. NLPHL cases with transformation into pure THRLBCL were not included in the present study, since different mechanisms of transformation may be involved in these cases.

In a second step, 41 consecutive cases of male patients with DLBCL, diagnosed at the Pathology Department of Tampere University between 2003 and 2010, were selected from the archives and analyzed to identify cases representing a transformation from NLPHL (“LP-type DLBCL”).

The study was approved by the local ethics committees of Frankfurt and Tampere University Hospital (ethics votes 120/13 from May, 23rd 2013 and 7085/05.01.00.06/2010 from October, 5th 2010).

### Immunohistochemistry

All composite cases of NLPHL and DLBCL were investigated for CD20, CD79a, CD19, CD3, EMA, J-chain, CD75, CD10, BCL2, BCL6, CD30, CD15, MUM1, IgD, p-STAT6, JAK2 and EBER.

The cases of the second series of DLBCL were stained for CD20, CD3, CD10, BCL6, BCL2, CD75, EMA, J-chain and MUM1. In cases where transformation from NLPHL seemed likely, the specimens were additionally studied for IgD, CD30, CD15, and EBER. Twenty-eight cases each of DLBCL derived from NLPHL and conventional DLBCL were evaluated for the presence of follicular dendritic cells (FDCs) by CD21 and CD23.

Immunostainings were performed in the Pathology departments of Frankfurt and Tampere University using either an Alkaline Phosphatase-Real Detection Kit (DAKO, Glostrup, Denmark) or Peroxidase-EnVision Plus Kit (DAKO) as described previously [20]. The antibodies used, dilutions, and providers are listed in Additional file 1: Table S1. Antigen unmasking was performed for 3 min in a pressure cooker in TRIS-EDTA pH 8.0. All stainings were assessed using a multi-head microscope, and were scored as positive or weakly positive if > 50% of the tumor cells showed a reaction with the respective antibodies. Positivity perceivable at low magnification (4×) was scored strongly positive, positivity only noticeable at higher magnification was scored weakly positive. Intensity of B cell markers (CD20, CD79a, CD19) was scored weakly positive if the expression intensity was weaker than in small reactive B cells. In the composite cases it was also assessed, if the tumor cells in the DLBCL component showed enhanced or attenuated expression compared with the LP cells in the NLPHL component.

## Results

### Clinical and morphologic findings of NLPHL + DLBCL composite lymphomas

Twenty-six patients presented with NLPHL and DLBCL simultaneously in the same lymph node. Seven patients developed sequential DLBCL secondary to the previous diagnosis of NLPHL with a median time to transformation of 8 years. The median patient age (50 years) did not differ between patients with simultaneous or sequential presentation of the disease. Detailed clinical characteristics are displayed in Table 1. In twenty patients, the treatment and follow up was known: 65% of the patients were treated by R-CHOP, 10% received ABVD, 10% did not receive any treatment due to poor performance status. One patient received rituximab only, and two patients underwent autologous stem cell transplantation. The median follow up time in these 20 patients was 21 months (21.5 months for alive patients), and the estimated 8-year progression free survival (PFS) and overall survival (OS) rates were 69% and 54%, respectively. Patients with sequential transformation showed a non-significant trend towards worse overall and progression free survival compared to patients with simultaneous presentation of NLPHL and DLBCL (Additional file 2: Figure S1).

**Table 1:** Clinical characteristics of patients studied

	**NLPHL-DLBCL (n =** **33)**	**Conventional DLBCL (n =** **41)**
**Patients with simultaneous involvement by NLPHL and DLBCL**	**26**	**-**
**Patients with sequential involvement by NLPHL and DLBCL**	**7**	**-**
**Median patient age (range)**	**50 (19–78)**	**69 (19–93)**
**male gender (%)**	**85**	100*
**Biopsy site**		
**- axillary**	**12 (36%)**	**4 (10%)**
**- cervical/supraclavicular**	**9 (27%)**	**26 (63%)**
**- abdominal/mesenteric LN/bowel**	**8 (24%)**	**5 (12%)**
**- inguinal**	**4(12%)**	**1 (2%)**
**- mediastinal/retroperitoneal**	**0**	**3 (7%)**
**- other**	**0**	**2 (5%)**
**Splenic involvement**	**30%**	**5%**
Stage**		
**- I**	**2 (9%)**	**7 (17%)**
**- II**	**4 (18%)**	**12 (29%)**
**- III**	**6 (27%)**	**12 (29%)**
**- IV**	**10 (45%)**	**10 (24%)**

*only male patients were selected.

**Clinical stages were available in 22 of 33 NLPHL-DLBCL composite lymphomas.

### Morphologic findings of NLPHL + DLBCL composite lymphomas

**Figure 1:**
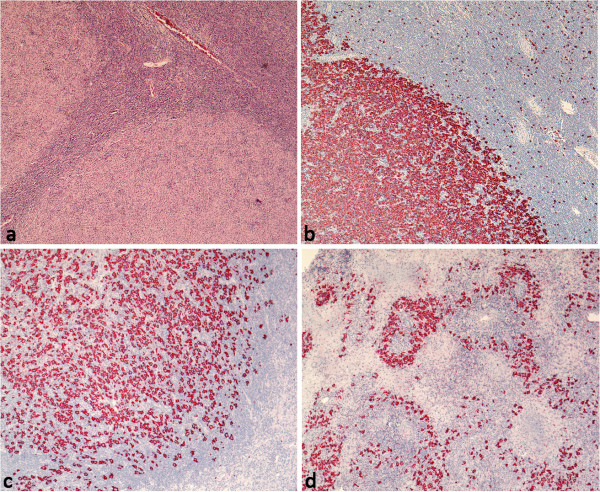
**Typical infiltration patterns of** “**LP type**” **DLBCL in composite lymphomas. a**. Sharply demarcated infiltrate of pale staining blasts, HE (40×). **b**. Sharply demarcated blast infiltrate, CD20 (40×). **c**. Sharply demarcated blast infiltrate with T cell and histiocyte rich microenvironment (CD20, 40×). **d**. DLBCL with abundant necrosis and blast infiltrate predominantly in perivascular localization (CD20, 40×).

The ratios of the NLPHL and DLBCL components were heterogeneous in the infiltrated tissue. In some cases, only a few nodules of NLPHL could be observed at the margins of the tissue effaced by the DLBCL. The NLPHL component showed a typical nodular pattern in 10 cases (pattern A according to Fan *et al*. [4]); whereas, 23 cases presented T cell rich and diffuse patterns (patterns non-A/B). DLBCL transformed from NLPHL consisted either of nodular, sharply demarcated, large cohesive sheets of blasts with few bystander cells (21 cases, Figure 1a and b) or of small sheets of blasts, with an abundant T cell and histiocyte infiltrate (12 cases, Figure 1c and d). These latter cases were reminiscent of T cell/histiocyte rich large B cell lymphoma, but the tumor cell content was greater than 10% of the total infiltrate and the blasts formed small clusters. Whereas necrosis was never observed in NLPHL, four cases showed necrotic foci within the DLBCL component. In these areas, the blasts showed a predominant perivascular distribution (Figure 1d). In all cases the blasts exhibited large nuclei compared with common de novo DLBCL, and presented a broad, pale cytoplasm. The morphologies of the nuclei were variable (12 centroblastic polymorph, 11 multilobated, 6 anaplastic, 3 centroblastic monomorph, 1 immunoblastic).

### Immunophenotype of NLPHL + DLBCL composite lymphomas

Immunostainings were scored positive, weakly positive, or negative in the tumor cells of both NLPHL and DLBCL components. The intensity of the staining of both components was compared and was also recorded if enhanced or reduced expression in the DLBCL component was observed (Table 2).

**Table 2:** Immunophenotype of composite lymphomas with NLPHL and DLBCL

**Immuno-staining**	**DLBCL component positive**	**LP cells positive**	**Reduced expression in DLBCL component**	**Enhanced expression in DLBCL component**	**Identical expression in DLBCL and NLPHL component**	**Identical expression in DLBCL and NLPHL in simultaneous cases**
Bcl6	33/33 (100%)	33/33 (100%)	3%	0%	97%	96%
CD79a	33/33 (100%)	33/33 (100%)	0%	33%	67%	62%
CD19	32/33 (97%)	25/32 (78%)	3%	66%	31%	32%
CD75	31/33 (94%)	32/33 (97%)	12%	9%	79%	81%
EMA	24/33 (73%)	27/33 (82%)	18%	6%	76%	85%
J-chain	24/33 (73%)	27/33 (82%)	27%	3%	70%	77%
Bcl2*	20/32 (63%)	27/33 (82%)	0%	38%	63%	69%
MUM1	21/33 (63%)	24/32 (72%)	13%	13%	75%	77%
JAK2	12/30 (40%)	4/27 (15%)	0%	23%	77%	82%
CD10	12/33 (36%)	2/33 (6%)	0%	33%	67%	69%
p-STAT6	9/30 (30%)	8/26 (31%)	9%	5%	86%	89%
CD30	7/33 (21%)	7/33 (21%)	6%	6%	88%	92%
CD15	4/33 (12%)	2/33 (6%)	0%	12%	88%	85%
IgD	4/33 (12%)	5/33 (15%)	3%	0%	97%	96%

Immunohistochemical stainings were assessed separately in the NLPHL and DLBCL component. Stainings were also assessed if there was an enhanced or decreased staining intensity in the DLBCL component compared to the LP cells in NLPHL.

*Since BCL2 was usually negative or weakly positive in LP cells, the frequency of negative or weakly positive cases is given.

**Figure 2:**
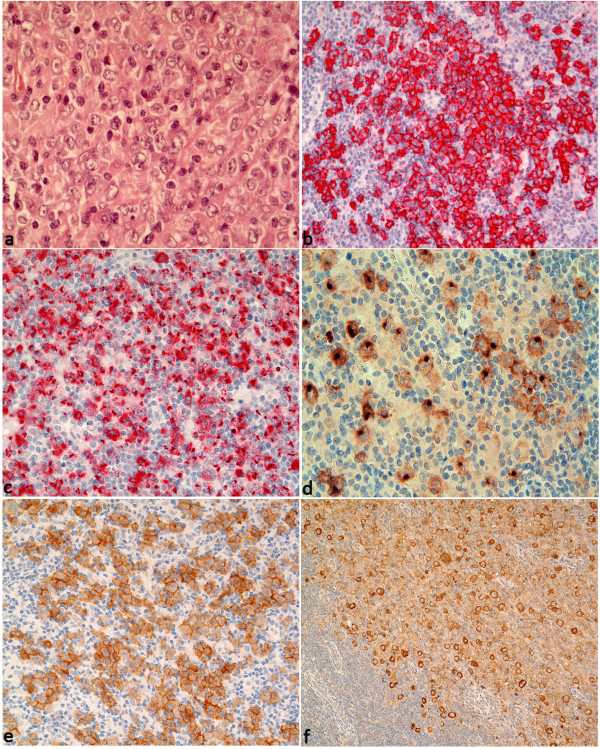
**Typical and special features of** “**LP type**” **DLBCL in composite lymphomas. a**. Typical blast infiltrate with broad pale cytoplasm and lobulated LP cell-like nuclei (HE, 400×). **b**. Blast infiltrate strongly expressing EMA as observed in 73% of “LP type” DLBCL (EMA immunostaining, 200×). **c**. Expression of CD15 in the cytoplasm and Golgi field in 4 of 33 “LP type” DLBCL (CD15 immunostaining, 200×). **d**. Expression of CD10 in the cytoplasm and Golgi in 36% of “LP type” DLBCL (CD10 immunostaining, 400×). **e**. IgD expression in blasts observed in 4 of 33 “LP type” DLBCL (IgD immunostaining, 200×). **f**. JAK2 expression found in 43% of “LP type” DLBCL (JAK2 immunostaining, 100×).

**Figure 3:**
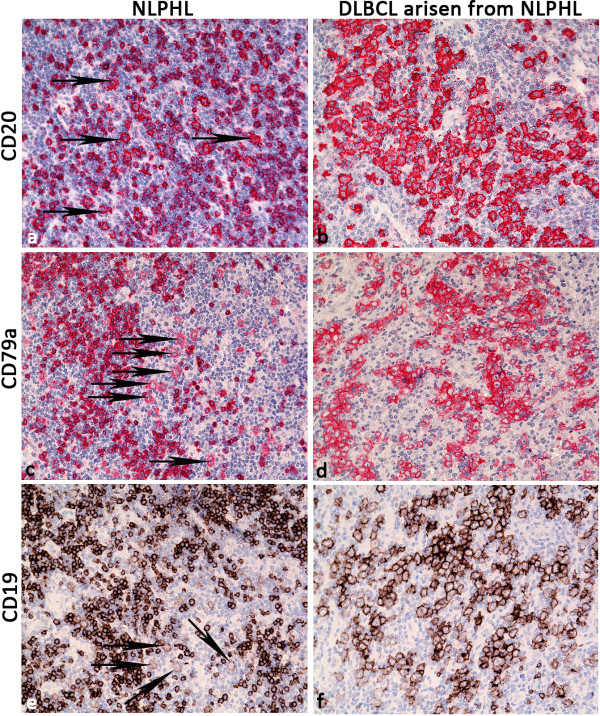
**B cell phenotype in composite lymphomas of NLPHL and** “**LP type**” **DLBCL. a**. Moderate CD20 expression in LP cells of NLPHL (arrows: CD20, 200×). **b**. More enhanced CD20 expression in “LP type” DLBCL (same lymph node and same section, CD20, 200×). **c**. Weak CD79a expression in LP cells of NLPHL (arrows: CD79a, 200×). **d**. More enhanced CD79a expression in “LP type” DLBCL (same lymph node, CD79a, 200×). **e**. Weak CD19 expression in LP cells of NLPHL (arrows: CD19, 200×). **f**. More enhanced CD19 expression in “LP type” DLBCL (same lymph node, CD19, 200×).

The immunophenotype of neoplastic cells in both the NLPHL part and the DLBCL components were largely consistent with the immunophenotype previously reported for LP cells [1]. However, 32 of 33 cases showed a difference in the immunophenotype or intensity between NLPHL and DLBCL in at least one staining. BCL6 and CD79a were expressed in the tumor cells of both components in all cases. CD75, EMA, and J-chain were expressed in most cases in the LP cells of NLPHL (97%, 82%, and 82% of the cases, respectively, Table 2, Figure 2) and in the tumor cells of the DLBCL component (94%, 73%, and 73% of the cases, respectively). Although attenuation or loss of expression of EMA and J-chain was observed in the DLBCL component, these markers were significantly more frequently positive compared with conventional DLBCL (p < 0.05, 2-Tail Fisher´s Exact Test, see below). MUM1 was weakly expressed as is typically observed in germinal center B cells [21] at approximately the same frequency in both the NLPHL and DLBCL components (63% and 72%). Surprisingly, B cell markers were more likely to exhibit enhanced expression in the DLBCL component: CD19 and CD79a were more strongly expressed in the DLBCL component compared to the LP cells in 66% and 33% of the cases, respectively (Table 2, Figure 3). The LP cells were negative or only weakly positive for BCL2 in 82%. However, BCL2 and CD10 (Figure 2d) were upregulated in the DLBCL component in 38 and 33% of cases, respectively. JAK2, p-STAT6, CD30, CD15, and IgD were expressed in only a few cases of both DLBCL and NLPHL. There was no correlation between the JAK2- and p-STAT6-positive cases. The four cases strongly expressing CD15 (>80% of the tumor cells) in the DLBCL component were CD30-negative. All cases were negative for EBER.

### Independent series of conventional DLBCL

In order to compare DLBCL transformed from NLPHL (“LP type” DLBCL) with conventional DLBCL and to identify more cases that may be derived from NLPHL, we evaluated an independent series of 41 DLBCL for their morphology and immunophenotype. Because of the high prevalence of males in the first series, we decided to investigate all males diagnosed with DLBCL at the Pathology Department of Tampere between 2003 and 2013. Clinical data are shown in Table 1. Because we had observed typical positivity for BCL6, CD75, EMA, J-chain, and weak MUM1 expression as well as negativity or weak positivity for BCL2 in “LP type” DLBCL, all cases of the second series were stained for theses antigens and were additionally evaluated for the presence of FDCs.

**Figure 4:**
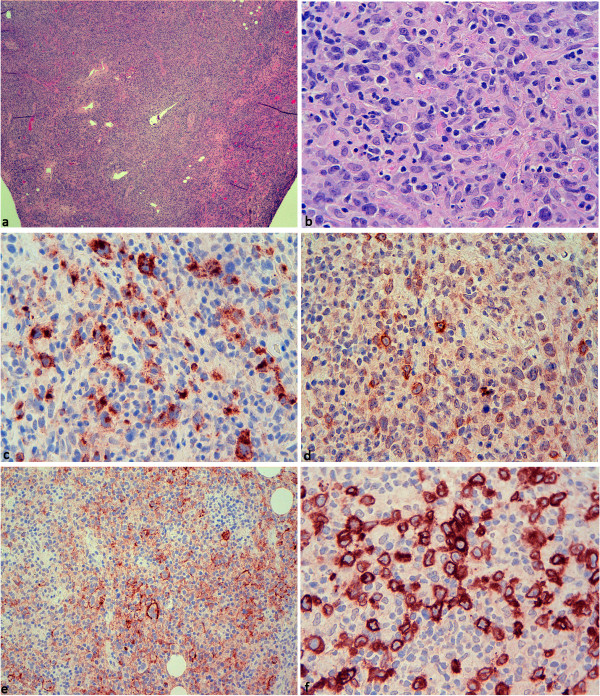
“**LP type**” **DLBCL**, **presumably transformed from NLPHL.** Cervical lymph node biopsy of one of the patients presenting with “LP type” DLBCL. **a**. HE, 40×. The lymph node architecture is effaced with open sinuses. **b**. HE, 400×. Higher magnification reveals a blast infiltrate with a broad, pale cytoplasm and large numbers of histiocytes. **c**. Blasts stain partly positive for CD75 (400×). **d**. A subset of blasts is positive for J-chain (400×). **e**. The infiltrate weakly expresses EMA (200×). **f**. IgD is strongly expressed in the blast population (400×).

All cases were positive for CD20, and the majority of cases were positive for BCL6, CD75, and MUM1 (83%, 85%, and 83%, respectively, Table 3). FDCs were rarely observed in both “LP type” and conventional DLBCL (Table 3). However, EMA (27%) and J-chain (22%) were significantly less frequently positive (p < 0.05, 2-Tail Fisher´s Exact Test) in conventional DLBCL compared to “LP type” DLBCL. Only three cases expressed both EMA and J-chain (see below). Likewise, a negative or weakly positive BCL2 staining was only observed in 32% of cases of conventional DLBCL in the second series and was significantly less frequently observed than in “LP type” DLBCL (p < 0.05, 2-Tail Fisher´s Exact Test). Therefore we selected all cases with a nodular, sheet-like growth pattern or a histiocyte rich microenvironment and expression of BCL6, CD75, and J-chain and (or) EMA. Five cases meeting these criteria, including the three EMA-J-chain double positive cases, were further evaluated for CD30, CD15, EBER, IgD, and CD19. One of these sheet-like growing DLBCL, which was positive for BCL6, CD75, J-chain, and EMA and involved the parotid gland of a 36 year-old male, could be excluded as a transformed marginal zone lymphoma: after closer work-up of this case, a low-grade lymphoma component with a typical follicular colonization pattern was discovered in an additional tissue block. Finally, four “LP type” DLBCL were identified among the 41 DLBCL cases (10%, Figure 4). All four cases were positive for BCL6, CD75, and EMA. Three cases were additionally positive for J-chain and weakly positive or negative for BCL2. One case was positive for IgD, one for CD10, and one for CD30. All four cases were negative for EBER and CD15.

**Table 3:** **Immunophenotype of** “**LP type**” **DLBCL** (**transformed from NLPHL**) **compared to conventional DLBCL**

**Staining pattern typically observed in DLBCL transformed from NLPHL**	**“LP type” DLBCL (n =** **33)**	**Conventional DLBCL (n =** **41)**
Bcl-6 strongly positive	33 (100%)	34 (83%)
CD10 weakly or strongly positive	12 (36%)	15 (37%)
CD75 weakly or strongly positive	31 (94%)	35 (85%)
EMA weakly or strongly positive	24 (73%)*	11 (27%)*
MUM1 weakly or strongly positive	21 (63%)	34 (83%)
J-chain weakly or strongly positive	24 (73%)*	9 (22%)*
Bcl2 weakly positive or negative	20 (63%)*	13 (32%)*
CD21-positive follicular dendritic cells	1/28 (4%)	5/28 (18%)
CD23-positive follicular dendritic cells	0/28 (0%)	5/28 (18%)

*Staining patterns which were significantly more frequently observed in “LP type” DLBCL compared to conventional DLBCL are printed in bold (p < 0.05, 2-Tail Fisher´s Exact Test).

### Clinical features of four DLBCL cases with putative derivation from NLPHL

The median age of these patients was 31.5 years. The localization of the biopsies were cervical (in two cases), axillary and mesenteric. One patient was diagnosed in stage II, one in stage III, and two in stage IV. Three of four patients presented splenic involvement, and two of these additionally presented liver involvement. Two patients achieved complete remission after R-CHOP/R-CHOEP, and one patient achieved remission after R-CHOP and subsequent autologous stem cell transplant. One patient received DHAP but died few months after diagnosis.

## Discussion

In the present study we investigated 33 patients with NLPHL and transformation into DLBCL. Although 26 of 33 patients in the present study showed a simultaneous presentation of NLPHL and DLBCL, the clinical data match well with previous studies of secondary transformations of NLPHL into DLBCL [16,17]. Histopathologic variant patterns (“Non-A/B”) as described by Fan *et al*. [4] were seen in the NLPHL component in 70% of composite cases investigated in the present study. Histopathologic variants have been associated with advanced stage and an increased risk of relapse [4,5,22] and the high frequency in the present study likely points to an increased risk of transformation into DLBCL in these patients.

In the present study, we observed in most composite NLPHL-DLBCL cases, a particular pattern of infiltration with a sharply demarcated sheet-like blast expansion or with a relatively high number of histiocytes admixed with the DLBCL component. The morphology of the nuclei was variable, as previously observed [14]. The most common immunophenotype, differing from conventional DLBCL, was coexpression of EMA and J-chain. However, this immunophenotype was not specific. EMA expression in DLBCL derived from NLPHL was also observed in one previous study [14]. Another remarkable observation was the upregulation of B cell markers as well as BCL2 in the DLBCL component compared to the NLPHL component. Possibly, the slight downregulation of B cell receptor signaling observed in the LP cells of NLPHL [6,8] is substituted by stimulation from rosetting follicular T helper cells. Since T cell rosetting was never observed in the DLBCL component, these tumor cells must have acquired additional properties to ensure their survival. One of these features may represent enhanced tonic active B cell receptor signaling [23-25] or the upregulation of BCL2 anti-apoptotic protein [26,27]. The increased number of tumor cells in “LP type” DLBCL compared to the NLPHL component may result from enhanced anti-apoptotic properties resulting from BCL2 expression. In the present series, p-STAT6 and JAK2 were expressed in both components less frequently than previously observed [9]. Possibly, SOCS1-mutated NLPHL cases with active JAK-STAT-signaling are less likely to transform into DLBCL and may, therefore, have a better prognosis similar to DLBCL with truncating SOCS1 mutations [28]. We also observed upregulation of CD15 and CD10 in some cases of “LP type” DLBCL. CD15 expression has been described in a subset of NLPHL-DLBCL composite lymphomas [14], whereas CD15 expression in NLPHL generally is a rare phenomenon [3,29,30]. Upregulation of these markers may be associated with activation of the tumor cells as observed for CD30 expression [31-33].

Since we also identified four cases with a typical “LP type” infiltration pattern and immunophenotype in a series of conventional DLBCL, we hypothesize that a series of conventional DLBCL cases will include cases with transformation from NLPHL, particularly if diagnoses are made on small core biopsies. Since some of the composite cases showed a downregulation of J-chain and EMA as well as an upregulation of CD10 and BCL2 in the DLBCL component, these cases may be difficult to identify applying current histopathologic methods if only the DLBCL component is sampled. Furthermore, the immunohistochemical stainings, observed to be frequently positive in the present study, were not specific, as was demonstrated in the case of transformed marginal zone lymphoma, which was positive for both EMA and J-chain.

## Conclusions

In the present study we observed a heterogeneous immunophenotype of DLBCL derived from NLPHL, which may reflect different mechanisms of transformation. This fact makes these cases difficult to recognize, if the NLPHL component is not sampled. Men with sharply demarcated blast infiltrates with EMA- and/or J-chain expression and abdominal, splenic, or axillary localization are more likely to have “LP type” DLBCL. However, it is important to state, that by applying current immunohistochemical markers, there is no way to specifically recognize these cases unless the coexisting NLPHL component can be identified. Therefore, further characterization of the particular clinical and molecular features of “LP type” DLBCL among conventional DLBCL is warranted, and may lead to identification of better diagnostic markers.

## Competing interests

The authors report no potential conflict of interest.

## Authors’ contributions

SH and MV: design of the study, acquired and analysed data, drafted the manuscript; ME, UB, TL, PK: acquired and analysed data; CD: biostatistical analysis; MLH: design of the study and drafted the manuscript. All authors read and approved the final manuscript.

## Pre-publication history

The pre-publication history for this paper can be accessed here:

http://www.biomedcentral.com/1471-2407/14/332/prepub

## Supplementary Material

Additional file 1: Table S1 Antibodies, dilutions and providers applied in the study. Click here for file

Additional file 2: Figure S1 Progression free survival (PFS) and overall survival (OS) of patients with NLPHL and transformation into DLBCL. **a.** Kaplan-Meier-analysis of overall survival of patients with simultaneous and sequential presentation of NLPHL and DLBCL. **b.** Kaplan-Meier-analysis of progression free survival of patients with simultaneous and sequential presentation of NLPHL and DLBCL. Click here for file
