# Supplementary material for: Diffuse large B cell lymphoma derived from nodular lymphocyte predominant Hodgkin lymphoma presents with variable histopathology
Source: BMC Cancer. 2014 May 13;14:332. doi: 10.1186/1471-2407-14-332 (PMC4030276; doi:10.1186/1471-2407-14-332)
Supplement: Additional file 1: Table S1 — Antibodies, dilutions and providers applied in the study. [file 1471-2407-14-332-S1.doc]

**Additional file 1: Table 1.** Antibodies, dilutions and providers applied in the study.

* These stainings were performed as described previously in Mottok et al.^1^

|  | Dilution | Company |
| --- | --- | --- |
| CD20 | 1:1000 | Dako, Glostrup, Denmark, M0755 |
| CD79a | Ready to use | Dako, IR 621 |
| CD19 | Ready to use | Dako, IR 656 |
| CD3 | 1:200 | Novocastra, Newcastle upon Tyne, UK, NCL-CD3-PS1 |
| EMA | Ready to use | Dako, IR 629 |
| J-chain | 1:100 | DCS, JC88 |
| CD75 | 1:1000 | Abcam |
| CD10 | Ready to use | Dako, IR 648 |
| BCL2 | Ready to use | Dako, IR 614 |
| BCL6 | 1:25 | Dako, M7211 |
| CD30 | 1:100 | Dako, M0751 |
| CD15 | Ready to use | Dako, IR 062 |
| MUM1 | 1:200 | Dako, M7259 |
| IgD | 1:200 | Novocastra |
| p-STAT6 | 1:1000 | B&D Biosciences, San Diego, CA, 611566* |
| JAK2 | 1:50 | Cell Signaling, Beverly, MA, 24B11 * |
| CD21 | 1:100 | Dako, M0784 |
| CD23 | Ready to use | Dako, IR781 |

**Reference**

1. Mottok A, Renne C, Willenbrock K, Hansmann ML, Brauninger A. Somatic hypermutation of SOCS1 in lymphocyte-predominant Hodgkin lymphoma is accompanied by high JAK2 expression and activation of STAT6. Blood. 2007;110:3387-3390.
