# Supplementary figures and images for: Diffuse large B cell lymphoma derived from nodular lymphocyte predominant Hodgkin lymphoma presents with variable histopathology
Source: BMC Cancer. 2014 May 13;14:332. doi: 10.1186/1471-2407-14-332 (PMC4030276; doi:10.1186/1471-2407-14-332)

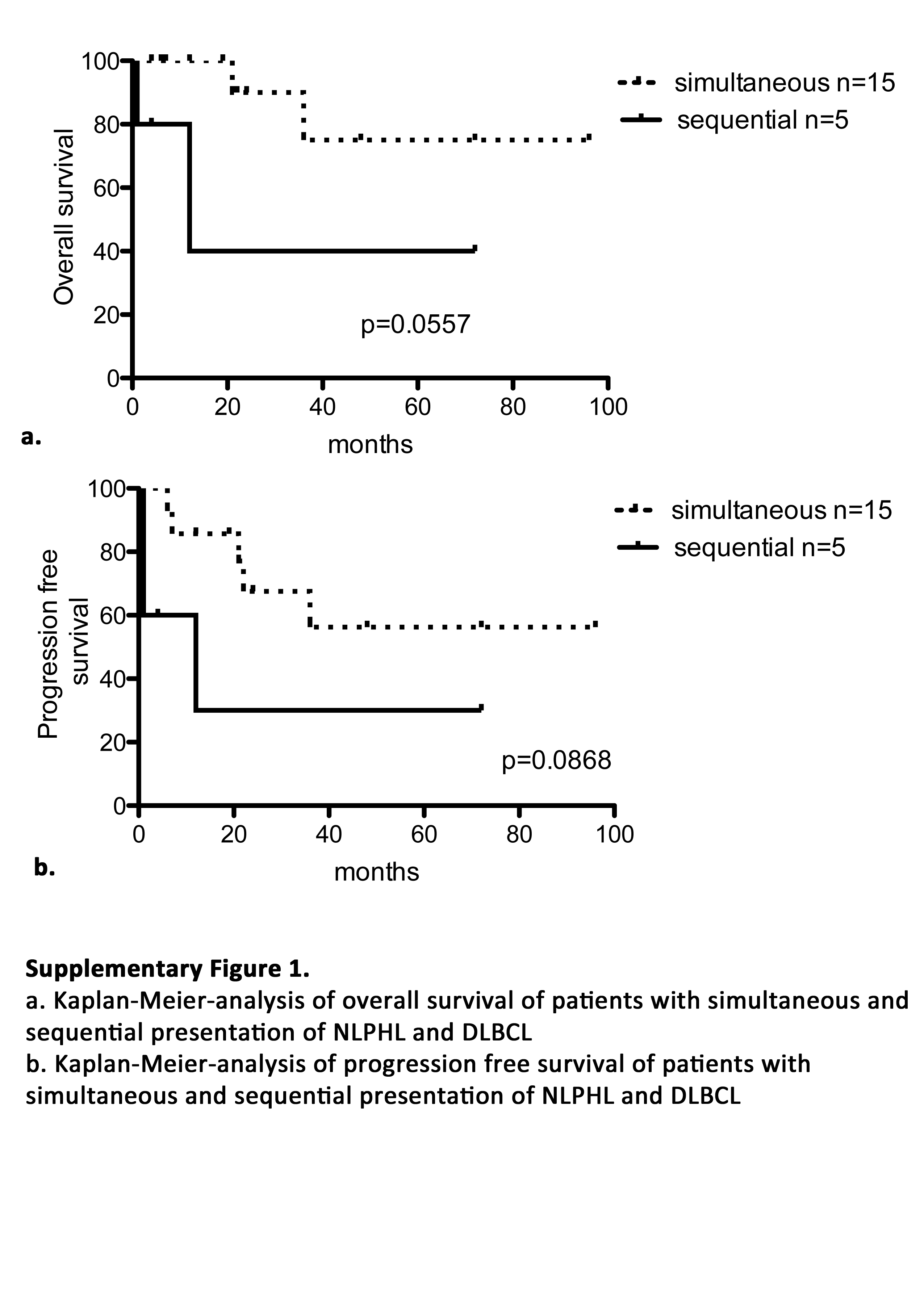

Supplement: Additional file 2: Figure S1 — Progression free survival (PFS) and overall survival (OS) of patients with NLPHL and transformation into DLBCL. a. Kaplan-Meier-analysis of overall survival of patients with simultaneous and sequential presentation of NLPHL and DLBCL. b. Kaplan-Meier-analysis of progression free survival of patients with simultaneous and sequential presentation of NLPHL and DLBCL. [file 1471-2407-14-332-S2.jpeg]
